# Paraphrenia revisited: psychotic states arising later in life. Why do psychiatrists tend to overlook it?

**DOI:** 10.1192/j.eurpsy.2023.2304

**Published:** 2023-07-19

**Authors:** R. M. Sousa, S. Martins, J. Correia, N. Cunha

**Affiliations:** 1 Centro Hospitalar Tondela-Viseu, Viseu; 2 Centro Hospitalar Tâmega e Sousa, Penafiel; 3 Unidade de Saúde Local da Guarda, Guarda, Portugal

## Abstract

**Introduction:**

In spite of the progress observed in the last decade particularly in the field of the neurosciences, areas of controversy and incomplete concepts still remain in psychiatry. One relates to the study the heterogeneous group of schizophrenic spectrum functional psychosis that arise along the neurophysiological aging process. Kraepelin first used the term paraphrenia in 1912, to describe a psychotic disorder with much lighter impairment of emotion and volition, minimal to no cognitive deterioration (dementia) and personality preservation compared to *dementia praecox.* However, since its first descriptions, late-onset psychoses have received different descriptions and definitions.

**Objectives:**

Brief review of the evolution of paraphrenia concept, focusing not only on pioneering currents, but also articulating it with recent conclusions on late-onset psychoses.

**Methods:**

Systematic revision of literature.

**Results:**

After Kreapelin pioneerism, Bleuler and Mayer-Gross would contribute to the weakening and disruption of the *Kraepelinian* concept of paraphrenia. In the first half of the 20th century, psychiatry was moving towards the dissolution of this concept. British psychiatrists would later rehabilitate the concept of paraphrenia but to designate a very late-onset variant of schizophrenia - late paraphrenia. This influenced the International Diseases Classifications (ICD), and the 8th edition was the first to consider paraphrenia as a subtype of paranoid schizophrenia.

By the end of the 20th century, both ICD-10 and various editions of DSM since DSM-III-TR (inclusive) omitted the category of paraphrenia, allowing the super-inclusiveness of the schizophrenia category and discouraging research on the theme.

In the late 20th century, late paraphrenia was conceived as a group of heterogeneous disorders that included paranoid and organic psychosis. To date, the term very late onset schizophrenia-like psychosis is the term used to replace late paraphrenia.

**Conclusions:**

The nosological consecration of paraphrenia suffered several misfortunes over the last century. The schizophrenic psychosis “black-hole” conceived at the same time contributed to this concealment. In addition, modern pharmacology also allowed the neuroleptization and homogenization of disorders with psychotic symptoms which led to the devaluation of some diagnostic possibilities in the “neighborhood” of schizophrenia.

We propose a nosological frame composed of two distinct entities: one based on a neurodevelopment disorder - schizophrenia - with insidious onset at a younger age, with a hereditary background and greater global deterioration, an the other, with a neurodegenerative basis - paraphrenia - with an abrupt and later onset, less contribution of genetic factors, greater preservation and lower probability of dementia development.

**Disclosure of Interest:**

None Declared

